# Bioimpedance Analysis in CKD and HF Patients: A Critical Review of Benefits, Limitations, and Future Directions

**DOI:** 10.3390/jcm13216502

**Published:** 2024-10-30

**Authors:** Edoardo La Porta, Alessandro Faragli, Alexander Herrmann, Francesco Paolo Lo Muzio, Luca Estienne, Stefano Geniere Nigra, Antonio Bellasi, Giacomo Deferrari, Giovanni Ricevuti, Salvatore Di Somma, Alessio Alogna

**Affiliations:** 1 UOC Nephrology, Dialysis and Trasplantation, IRCCS Istituto Giannina Gaslini, 16147 Genoa, Italy; 2 UOSD Dialysis, IRCCS Istituto Giannina Gaslini, 16147 Genoa, Italy; 3 Department of Cardiology, Angiology and Intensive Care Medicine, Deutsches Herzzentrum der Charité, Augustenburger Platz 1, 13353 Berlin, Germanyalessio.alogna@dhzc-charite.de (A.A.); 4 Charité-Universitätsmedizin Berlin, Corporate Member of Freie Universität Berlin and Humboldt-Universität zu Berlin, 10117 Berlin, Germany; 5 Berlin Institute of Health (BIH), 10178 Berlin, Germany; 6 DZHK (German Centre for Cardiovascular Research) Partner Site Berlin, 10785 Berlin, Germany; 7 Department of Cardiovascular Surgery, University Heart & Vascular Center Hamburg, University Medical Center Hamburg-Eppendorf, 20246 Hamburg, Germany; 8 Department of Nephrology and Dialysis, SS. Antonio e Biagio e Cesare Arrigo Hospital, 15121 Alessandria, Italy; 9 Emergency Department, AUSL Romagna M. Bufalini Hospital, 47521 Cesena, Italy; 10 Service of Nephrology, Ospedale Regionale di Lugano, Ospedale Civico, Ente Ospedaliero Cantonale, Via Tesserete 46, 6903 Lugano, Switzerland; 11 Department of Cardionephrology, Istituto Clinico Ligure di Alta Specialità (ICLAS), GVM Care and Research, 16035 Rapallo, GE, Italy; 12 Emergency Medicine, School of Pharmacy, University of Pavia, 27100 Pavia, Italy; 13 Department of Medical-Surgery Sciences and Translational Medicine, Sapienza University of Rome, 00184 Rome, Italy; 14 Great Network, Global Research on Acute Conditions Team, 00191 Rome, Italy

**Keywords:** bioimpedance analysis, chronic patients, heart failure, kidney disease, cardiology, nephrology

## Abstract

Bioimpedance analysis (BIA) is a validated non-invasive technique already proven to be useful for the diagnosis, prognosis, and management of body fluids in subjects with heart failure (HF) and chronic kidney disease (CKD). Although BIA has been widely employed for research purposes, its clinical application is still not fully widespread. The aim of this review is to provide a comprehensive overview of the state of the art of BIA utilization by analyzing the clinical benefits, limitations, and potential future developments in this clinically unexplored field.

## 1. Introduction

The human body behaves like a complex electric circuit [1,2]. This concept has been at the base of extensive research to understand how biological tissues respond to electrical stimuli and various models have been developed with the purpose to estimate the body composition [1,2,3,4]. A promising scientific area of research has emerged in the past 20 years, leading to the development of bioimpedance analysis (BIA) [3,4,5]. BIA exploits the set of electrical properties that characterize the opposition to an electric current flowing through biological tissue [3,4,5].

The main principle of BIA is based on building models that approximate the human body to an electric circuit with resistors and capacitors, studying the relationship between alternating electrical currents at different frequencies (i.e., single or multiple), body volume, and hydration status (HS) [3,6]. The raw measures of resistance (R), reactance (Xc) and phase angle (PA) are then employed to estimate the distribution of total body water (TBW) composition, comprising the measurements of intracellular water (ICW) and extracellular water (ECW), but also fat mass (FM) and fat free mass (FFM) [6,7,8]. Additionally, a set of hemodynamic parameters such as cardiac output and systemic vascular resistance can also be estimated with BIA [9].

BIA holds the potential to improve patient care, especially for conditions such as heart failure (HF) and end-stage kidney disease (ESKD), by supporting in the assessment of the health status (HS) of such patients [10,11]. In facts, even if the two conditions often exhibit a different etiology or initial presentation, their clinical course is similar, presenting recurrent decompensation events caused by episodes of congestion or at the opposite volume depletion [12]. The two diseases coexist in almost 40–60% of the cases, and in many situations, those patients are affected by the so-called cardiorenal syndrome (CRS), leading to poor prognosis and repeated unplanned hospitalizations [13]. In detail, CRS is a complex pathophysiological disorder involving the heart and kidneys in which either an acute or chronic dysfunction of one organ may induce the dysfunction of the other [13].

Cardiovascular diseases are the leading cause of mortality in chronic kidney disease (CKD) patients, reaching a 9-fold increased risk in hemodialysis (HD) patients compared to an age-matched general population [14]. Moreover, patients affected by CRS are more prone to worsening renal function and acute kidney injury (AKI) than CKD patients without cardiovascular disease [14,15].

Although BIA technology has been widely investigated, its application is still limited due to many factors and is far from being integrated into the clinical routine setting [12,16].

This narrative review was conducted to synthesize and evaluate the current literature on the clinical applications of BIA in HF and CKD. This review aimed to highlight studies that examined clinical endpoints such as mortality prediction, acute decompensation, and other hard clinical outcomes in these patient populations, while also addressing the main clinical pitfalls of the BIA technique.

A comprehensive search of the scientific literature was conducted to identify relevant articles. The databases searched included PubMed. The search terms used were combinations of the following: “bioimpedance analysis”, “heart failure”, “chronic kidney disease”, “end-stage renal disease”, “cardiorenal syndrome”, “mortality prediction”, “fluid overload”, and “decompensation.” Additional keywords related to BIA technologies, such as “single-frequency BIA”, “multiple-frequency BIA”, and “BIVA”, were also included. The search included articles published between 1980 and 2024. Studies were selected based on their relevance to the clinical application of BIA in HF and CKD.

The inclusion criteria for this review were as follows: original research articles, clinical trials, and meta-analyses that evaluated the use of BIA in HF or CKD patients and studies that reported on clinical outcomes such as mortality, hospitalizations, fluid overload, hemodialysis therapy and the management of diuretics. Articles were excluded if they focused on animal or in vitro studies or lacked sufficient data on the clinical application of BIA in these populations.

Data extraction was performed for each study to capture key information, including the study design, patient population, the specific BIA methodology employed (e.g., single-frequency BIA, multiple-frequency BIA, BIVA, BIS), and the clinical outcomes reported. The main findings of each study were summarized and synthesized to identify important themes and trends.

The review followed a narrative synthesis approach, focusing on a qualitative analysis rather than a formal systematic review. Studies were organized according to the clinical outcomes they evaluated, including mortality prediction, fluid overload management, and rehospitalization rates. The aim was to highlight the trends, gaps, and limitations in the current literature, while also discussing the clinical benefits and drawbacks of using BIA for managing patients with HF and CKD.

Despite the strengths of this review, certain methodical limitations must be acknowledged. As a narrative review, there was potential for bias in the selection of studies, and the absence of a systematic methodology for study inclusion may have impacted the comprehensiveness of the review. Additionally, since a meta-analysis was not conducted, this review did not provide a quantitative comparison of the results across different studies.

Therefore, our review will not focus in detail on the technical aspects and methodologies of BIA as this information has been previously and extensively published [10,11,17].

Finally, we aim to identify what could be improved or changed considering future paths of development in the field.

## 2. BIA Methods

BIA techniques differ according to the number of frequencies employed, being either single- or multiple-frequency methods, and they are commonly classified into single frequency (SF-BIA), generally measured at 50 kHz, and multiple-frequency (MF-BIA), ranging between 5 and 1000 kHz [11]. An in-depth description of these methods is beyond the scope of our narrative review; therefore, in this section, we will briefly describe the main concepts to highlight the technical advantages and disadvantages of the techniques.

### 2.1. Single-Frequency BIA (SF-BIA) and BIVA

SF-BIA is based on the inverse relationship between the measured impedance and TBW, which serves as the conductive pathway for the electric current [11].

Among the SF-BIA method, the bioimpedance vectorial analysis (BIVA) approach has attracted the attention of clinicians due to the ability to accurately classify the hydration status (expressed as normohydration or overhydration) [3]. By measuring the R and Xc of the current, BIVA can generate a vector in a two-dimensional impedance plane [3], which is then analyzed in respect to population-specific reference ellipses to infer information about an individual’s body composition and hydration status [3]. The process is non-invasive, quick, and can be performed in various settings, making it a versatile tool for the diagnosis and risk stratification of chronic and acute patients at higher risk of dehydration [3].

### 2.2. Multiple-Frequency BIA (MF-BIA) and BIS

The examination of BIA acquired at more than two frequencies is referred to as MF-BIA. This method relies on the observation that extracellular fluid (ECF) and TBW can be evaluated by subjecting them to low- and high-frequency electric currents, respectively [11].

In case 50 frequencies are applied, it is referred to as bioimpedance spectroscopy (BIS). The values obtained are translated onto a so-called Cole–Cole plot, allowing the extrapolation of resistances at a zero (R0) and infinite (R∞) frequency. This assumes that at the lowest frequencies, the current primarily flows through the ECF, while at high frequencies, it traverses ICW and ECW [11].

While SF-BIA is highly accurate for the assessment of TBW, for patients with a highly altered hydration status, MF-BIA has been shown to provide a more accurate prediction of ECF compared to the SF-BIA [17]. Nevertheless, among elderly individuals, the MF-BIA method exhibits reduced sensitivity in identifying fluid shifts between ECF and intracellular fluid (ICF) [17].

### 2.3. The Devices

In clinical practice, two BIA measuring methods have been primarily developed: the “external band electrode method” and the “implanted device lead method” [10]. The prevailing approach for measuring total body compartments is the hand-to-foot external band method [10], which was originally introduced by Hoffer and revised by Nyboer [18,19]. The approach aims to decrease the contact impedance between the skin and the electrodes and was validated for the first time by Lukaski on 140 healthy adults [2]. In a pioneering study, the authors avoided electrode polarization and reduced the effects of skin impedance by employing four electrodes: one pair for passing the current through the body, and the other to detect the resulting voltage [2]. In detail, the electrodes were placed on the dorsal side of the hands and feet, with a distance of at least 3–5 cm between the signaling and detection electrodes [2]. Tetra-polar measurements were taken on a supine subject, generally for 15 min, to allow the stabilization of the body fluids [2]. Ideally, before placing the electrodes, the surface should be cleaned with alcohol to avoid measurement bias [2].

Another way to utilize the external band electrode method is through thoracic BIA, which uses two pairs of external electrodes placed on the body surface on the thorax [20]. This method exploits the phenomenon whereby variations in fluid volume and blood flow determine changes in the frequency of propagating waves rather than in the amplitude of the signal [20,21]. Therefore, detecting the phase shift in electrical currents (known as bioreactance) also allows the assessment of cardiac output and vascular resistance [20,21]. The method is also known as impedance cardiography (ICG) [21].

A different method is based on the assessment of intrathoracic impedance by measuring the impedance between the device case, typically implanted in the left pectoral region, and the lead in the right ventricle [22]. Impedance has long been used to check for lead integrity in pacemakers or defibrillator devices, and intrathoracic bioimpedance measurements have also been developed and integrated into cardiac implantable electronic devices (CIEDs) to check volume status [23]. In fact, changes in impedance can be determined across two relatively fixed points, thus minimizing distortion, or via variations in electrode placement. Therefore, the primary purpose of implanted device-based BIA is to monitor over time the clinical status in chronically ill patients as an additional function of the implanted device [23,24].

Although BIA technology has been widely investigated, its application is, to this day, still limited due to many factors and is far from being integrated in the clinical routine setting [25].

In this article, we will refer generally to the term BIA for what concerns the bioimpedance analysis technology; nonetheless, we will specify for each study the type of technique (e.g., MF-BIA, BIVA, BIS) that has been used.

### 2.4. Clinical Relevance of BIA in HF Patients

HF presents the highest rate of hospitalizations among 65-year-old patients [26], with high morbidity and mortality rates, consuming vast resources of the public health system around the world [27]. The projected absolute number of people suffering from HF is approximately 26 million worldwide, and it is considered a growing epidemic with increasing incidence and prevalence [5,28]. In patients with acute decompensated HF (ADHF), body fluid congestion, presenting different clinical degrees of dyspnea, orthopnea, fatigue, jugular venous distension, rales, and edema, was demonstrated to be associated with re-hospitalization and high mortality rates [29]. Moreover, it is widely recognized that the pathophysiological determinant of the worsening of the patient’s state is caused by increased left ventricular filling pressures, the dysregulation of the effective circulatory volume, and consequent extracellular fluid retention and mostly intravascular congestion [30] that, if left untreated, leads to pulmonary edema [31]. It is known that approximately 90% of patients admitted to the emergency department exhibit signs of fluid overload [32], and as indicated by another study, about 50% of them are discharged with some degree of congestion [33].

When patients are admitted to hospital with excessive edema, intravenous diuretic therapy is commonly administered; however, since the monitoring is mostly performed through clinical observations, physical examination, or the evaluation of the diuresis, the overall body volume assessment is not precise [34,35].

Determining diuretic therapy efficacy also depends on the patient’s diuretic resistance, which often makes the in-hospital assessment of the body volume challenging. These difficulties are also quite expected in home-setting conditions [36].

In many peripheral wards, body weight changes are utilized to evaluate the performance of diuretic therapy, assuming that a deterioration of HF has been defined as an increase of 2 kg in 48/h [37]. Nonetheless, different studies have shown that body weight monitoring presents a low sensitivity for the timely detection of congestive events and does not completely correlate with the process of a patient’s decompensation [38,39].

Body volume assessment is critical at home since preventing acute decompensation events is an unmet necessity [40]. Therefore, many studies have investigated how to manage the volume status of HF patients by the time they leave the hospital to predict and avoid re-hospitalizations [37]. Although the cardioMEMS system, in which a small sensor is implanted the pulmonary artery to measure the pulmonary artery pressure, has been proven to be an effective method to prevent re-hospitalizations and reduce mortality in this set of patients, its use is limited by its invasiveness, costs, market penetration, and potential complications [41,42,43]. Non-invasive methods or devices, employed mostly for regularly checking the patient’s body weight and congestion symptoms, have been studied to promptly identify a potential heart failure decompensation event [44]. BIA has emerged to accurately detect the level of body fluids, and it has been studied as a potentially earlier predictor for decompensation events rather than an increase in body weight [29].

A selection of the most relevant clinical studies in HF patients studying hard endpoints that showed a beneficial or a neutral effect of BIA in both intra-hospital and homecare settings are summarized in Table 1.

One of the earliest studies on BIA, also known as the Prospective Evaluation and Identification of Cardiac Decompensation by ICG Test (PREDICT), was performed by Packer et al. to assess the feasibility of ICG in predicting clinical deterioration in ambulatory patients with HF [45]. In detail, the study prospectively evaluated 212 stable patients with chronic HF (CHF) who presented a recent episode of clinical decompensation; patients underwent clinical evaluation and blinded ICG testing every 2 weeks for 26 weeks and were followed up for the occurrence of death or hospitalization [45]. The study combined clinical parameters such as NYHA class and blood pressure with ICG parameters such as velocity index, thoracic fluid content (TFC) index, and left ventricular ejection time, producing a composite score. The score showed to predict with good accuracy an HF event during the next 14 days of follow-up (*p* = 0.0002). Moreover, visits with a high-risk ICG score presented an 8.4% event rate during the next 14 days and predicted 41.6% of the events [45].

In 2010, the group of Di Somma et al. aimed to verify whether BIVA could be a valid methodology for the assessment of fluid overload in ADHF, if there was a correlation with BNP, and if BIVA could be used in the management of the diuretic therapy in acute settings [46]. A total of 51 patients were enrolled, and their hydration state, BNP level, and caval index were evaluated at the following time points: at admission, after 24 and 72 h, and at discharge. Moreover, a follow-up by phone was performed after 3 months. BIOVA measured higher values of hydration state in ADHF patients compared to controls. The most interesting result was that patients with an average hydration value > 80.5% had a correlation with events at 3 months (death or rehospitalization for cardiogenic event) with a sensitivity of 22% and specificity of 94.2% [46].

In 2017, a prospective, multicenter, observational study by Santarelli et al. investigated the prognostic role of quantitative reduction in congestion during hospitalization by using BIVA serial evaluations in patients admitted for ADHF [47]. Both clinical and BIVA evaluations were performed at admission and discharge. A follow-up phone call was carried out at 90 days, and the primary endpoint was a composite of re-hospitalizations for HF or all-cause mortality. BIVA correctly predicted the primary endpoint at both admission (area under the curve (AUC) 0.56, *p* < 0.04) and discharge (AUC 0.57, *p* < 0.03). When combined with clinical evaluation, the prediction of the primary endpoint significantly increased for all-cause mortality or re-hospitalizations at 90 days (AUC 0.97, *p* < 0.0001). It is noteworthy that an increase in in-hospital resistance variation (dR/H) of more than 11 Ohm/m was associated with overall survival [47]. No significant results were found about the in-hospital reactance variation (dXc/H), underlining the concept that R is primarily influenced by changes in hydration status [47]. Furthermore, AHF patients discharged with the presence of at least one congestion sign were experiencing a lower survival [47].

BIA external devices have also been studied to assess their potential utilization for remote management in HF patients. The MUSIC study (Multisensor Monitoring in Congestive Heart Failure) was conducted to develop and validate an algorithm for predicting impending acute HF decompensation using a multi-parameter approach, including BIA obtained using an external device adhered to the chest [48]. This study found that the multisensor algorithms which included ICG as one of its sensors could accurately predict HF exacerbations with a sensitivity of 87% and a specificity of 87%. The algorithm also predicted HF aggravations an average of 6 days before clinical events occurred [48]. Moreover, BIA-derived measures of TFC were sensitive for predicting HF decompensation 9 to 11 days in advance. The study found that a TFC increase of 7.5% from baseline could predict clinical events with a sensitivity of 77% and a specificity of 64% [48].

Furthermore, in a prospective study by Gyllensten et al., 91 patients with CHF were monitored for an average of 10 months using a weight scale and a wearable BIA vest in home settings to predict HF exacerbations. One of the most important results was that BIA algorithms could better predict impending decompensation within 2 weeks compared to changes in weight (cross-validation estimate was 60% for BIA vs. 33% for body weight). However, the authors noted that many alerts detected through the BIA device and its algorithms were not associated with clinically overt decompensation and related HF hospitalizations [49].

The SENTINEL-HF study, instead, tested a wearable BIA device called a fluid accumulation vest. This device is non-invasive and capable of transmitting data via a mobile phone, and employs an automated algorithm to predict recurrent HF events. Among the study participants with sufficient data (*n* = 57), an algorithm analyzing thoracic BIA showed 87% sensitivity (95% CI 82–92), 70% specificity (95% CI 68–72), and 72% accuracy (95% CI 70–74) in the identification of HF events [50].

Different studies concentrated on analyzing intrathoracic congestion by applying BIA to the current generated from the pacing wires of pacemakers and defibrillators. One of the first studies on an ICD equipped with intrathoracic impedance monitoring was conducted by Wang et al. in a canine model [22,25,51]. The study demonstrated that impedance to electrical flow on the lead of these devices can reveal changes in thoracic congestion, mainly due to impending HF [22].

**Table 1:** Comparative analysis of BIA studies in HF patients. A selection of the studies exploring the hardest endpoints in HF patients is summarized in the table.

Author	Year	Type of Study	Patients	BIA Method	Endpoints	Limitations	Main Results
Packer- (PREDICT) [45]	2006	Prospective non-randomized study	212	Non-invasive transthoracic impedance	All-cause mortality or HF hospitalizations in 14 days	Small sample size	Beneficial Effects Clinical and ICG multi-parameters such as thoracic fluid content (TFC) predict HF at 14 days (*p* = 0.0002). High-risk ICG score presents an 8.4% event rate at 14 days (Accuracy: 41.6%)
Di Somma [46]	2010	Prospective non-randomized study	51	BIVA	All-cause mortality or HF hospitalizations in 90 days	Small sample size	Beneficial Effects Overhydration > 80.5% measured with BIVA correlated with primary endpoint at 3 months (Sensitivity: 22%; Specificity: 94.2%)
van Veldhuisen—(DOT-HF) [52]	2011	Randomized trial - Remote Monitoring	335	Intrathoracic impedance monitoring	All-cause mortality or HF hospitalizations in 15 months	Terminated prematurely due to slow enrollment	Neutral Effects No difference in mortality between groups (*p* = 0.54); number of outpatient visits was higher in the intervention group where intrathoracic impedance was measured (250 vs. 84; *p* < 0.0001).
Anand— (MUSIC Study) [48]	2012	Prospective non-randomized study - Remote Monitoring	543	Non-invasive transthoracic impedance	ADHF event in 90 days	High exclusion rate due to device failure or withdrawal of consent	Beneficial Effects TFC found sensitive for predicting HF decompensation up to 9–11 days in advance. TFC increase of 7.5% from baseline was found to predict the primary endpoint (Sensitivity: 77%; Specificity: 64%).
Gyllensten [49]	2016	Prospective non-randomized study - Remote Monitoring	91	Non-invasive transthoracic impedance	ADHF event in 14 days	Possibility of early intervention affecting results, small sample size	Beneficial Effects Non-invasive transthoracic BIA hydration status predicts a decompensation event 2 weeks in advance (*p* < 0.001). Neutral Effects Non-invasive transthoracic BIA and its algorithms had a low positive predictive value for overt HF decompensation events.
Darling— (SENTINEL -HF) [50]	2017	Prospective non-randomized study - Remote Monitoring	16	Non-invasive transthoracic impedance	ADHF event in 45 days	Small sample size, specificity was affected by false positives, homogeneous cohort	Beneficial Effects An algorithm utilizing multiparametric non-invasive thoracic BIA is highly predictive for the identification of recurrent HF events at 45 days (Accuracy: 72%)
Santarelli [47]	2017	Prospective non-randomized study	336	BIVA	All-cause mortality or HF hospitalizations in 90 days	Higher accuracy if BIVA is utilized with clinical signs	Beneficial Effects BIVA measured hydration status predicts the primary endpoints at admission (area under the curve (AUC) 0.56, *p* < 0.04) and at discharge (AUC 0.57, *p* < 0.03). By combining BIVA with clinical signs, a high predictive value for cardiovascular events at 90 days (AUC 0.97, *p* < 0.0001) was observed.
Anshory [53]	2024	Prospective non-randomized study	111	Non-invasive transthoracic impedance + BIVA	All-cause mortality or HF hospitalizations in 30 days	Drugs used in patient treatment and inotropic usage may have conditioned the results	Beneficial Effects Hemodynamic parameters like cardiac output, and TBW data significantly predicted 30-day cardiovascular mortality and rehospitalization. At discharge, a value of cardiac output was a significant predictor for 30-day rehospitalization.

ADHF = acute decompensated heart failure; AUC = area under the curve; BIA = bioimpedance analysis; BIVA = bioimpedance vectorial analysis; HF = heart failure; ICG = impedance cardiography; TFC = thoracic fluid content.

A study performed by Check-Man Yu et al. estimated that an implantable system capable of measuring intrathoracic impedance could identify fluid overload in patients before HF hospitalization and determine its correlation with standard measures of fluid status during hospitalization [25]. In detail, the automated detection of decreases in intrathoracic impedance via pacemaker and implantable cardioverter-defibrillator devices alerted in advance of the beginning of decompensation due to volume overload, providing timely information for titration of the medication [25]. Therefore, internal impedance-meter-equipped CIEDs have been developed with the product most known as Opti-Vol. In the DOT-HF trial, Opti-Vol affected the clinical outcomes of CHF patients, most likely due to additional data obtained from this method [52]. In the study, the number of deaths was comparable (*p* = 0.54), while the number of outpatient visits was higher in the access arm (250 vs. 84; *p* < 0.0001). Interestingly, the intervention was associated with a borderline statistically significant increase in the primary endpoint of all-cause mortality and HF hospitalizations. This increase was mostly due to increased HF-related admissions caused by the Opti-Vol method, which led to an increase in unneeded hospitalizations, thus not addressing the authors’ main hypothesis [52].

Eventually, a recent multi-centric study performed by Anshory et al. investigated the role of a non-invasive method to assess hemodynamic parameters and total body congestion via BIA (NICaS) [53]. In this work, the authors aimed to evaluate the above-mentioned technology in a cohort of Asian and Caucasian AHF patients predicting mortality and re-hospitalization for HF within 30 days. As a main result, data at admission significantly predicted 30-day cardiovascular mortality and rehospitalization. Moreover, at discharge, a value of cardiac output obtained using NICaS predicted significantly a 30-day rehospitalization. It is noteworthy that the total peripheral resistance index on admission and during 48–72 h showed each an AUC > 0.70 in predicting mortality and rehospitalization compared to the clinical congestion score and NT-proBNP. These findings underline the importance of the role of combined methodologies, such as total body water and hemodynamics, to better understand the patients’ clinical status.

### 2.5. Clinical Relevance of BIA in CKD Patients

Chronic kidney disease (CKD) is defined as a reduction in kidney function that persists for more than 3 months, with leading causes including diabetes and hypertension, as well as infectious glomerulonephritis, vasculitis, congenital anomalies of the kidney and urinary tract (CAKUT), and inherited and autoimmune diseases. Regardless of the etiology, CKD is characterized by progressive and accelerating loss of renal function caused by self-maintaining processes of fibrosis and atrophy of kidney tissues. Throughout the years, CKD can lead to end-stage kidney disease (ESKD), leading to potential kidney transplant or dialysis. The prevalence of CKD in the USA is as high as 40% among people over 70 years old, with most patients having CKD stage 3 (GFR 30–59 mL/min/1.73 m^2^) [54]. The main clinical manifestations are caused by the accumulation of uremic toxins, anemia, metabolic acidosis, malnutrition, altered electrolytes, and impaired water homeostasis, leading to volume expansion, hypertension, and peripheral/pulmonary edema. Chronic fluid overload is experienced by 40–50% of hemodialysis (HD) patients [54].

The volume status of a patient is a crucial parameter in the nephrology clinical practice, especially for those with concomitant renal and heart failure, in which both fluid overload and volume depletion events are frequent and are associated with CKD progression and poor outcomes [55,56].

A selection of the most relevant prospective clinical studies and metanalysis in CKD patients studying hard endpoints that showed a beneficial or neutral effect of BIA is presented in Table 2.

It has become clear that BIA (i.e., BIS) is the most widely used method to evaluate volume status and guide fluid management in nephrological patients [57,58]. In HD, BIS is already employed to assess the overhydration state, enabling clinicians to evaluate the amount of fluid that ultrafiltration should remove [57,58].

Hur et al. performed a well-structured randomized trial to investigate the effect of BIS-guided fluid management on cardiovascular outcomes in hemodialysis patients [59]. The authors used time-averaged fluid overload (TAFO) as a more accurate variable of the fluid status of patients. TAFO, calculated on pre-dialysis fluid overload (FO) and interdialytic weight gain (IDWG) (TAFO = FOpre − IDWG/2) was used to increase or decrease post-dialytic weight. The authors showed that bioimpedance fluid management significantly reduced the left ventricular mass index (LVMI) and improved systolic and diastolic blood pressure pre- and post-dialysis compared to standard care [59].

Onofriescu et al. showed in a randomized trial that a strict BIA-based (BIS) fluid management not only improved survival by significantly reducing mortality (hazard ratio (HR) = 0.100 (95% CI, 0.013–0.805; *p* = 0.03)), but also surrogated endpoints such as arterial stiffness, relative fluid overload, and systolic BP improved [60].

In another randomized trial, ABISAD-III researchers provided an algorithm to determine post-dialysis weight based on the evaluation of fluid status by a body composition monitor [61]. The incidence of acute fluid overload (AFO), cardiovascular events, hypertension, and intradialytic complications were significantly reduced in the intervention group [61].

Other works and specific meta-analysis studies have confirmed that excessive hydration defined by bioimpedance is an independent predictor of mortality in patients with ESKD [62,63].

A recent detailed methodological review and meta-analysis study on the use of whole-body BIA in dialysis patients was conducted on 46 manuscripts on the ESKD population; these studies were sub-grouped according to whether they used phase angle (PA)/BIVA, normalized ECW (ECW/TBW) or the overhydration index (OHI) [64]. The work evidenced that OHI is an independent predictor of mortality in ESKD patients independently of the influence of comorbidity and that different parameters, such as PA and OHI, act as similar predictors of outcome [64].

Another meta-analysis investigated the impact of bioimpedance-guided fluid management on two separated subsets of patients: CKD stage 3–5 and ESKD dialysis-dependent patients [65]. To our knowledge, this is the largest meta-analysis published on the BIA-based monitoring of fluid management in dialysis patients. The authors analyzed hard and secondary outcomes, and they showed a significantly reduced all-cause mortality (HR = 0.64, 95% CI: 0.41, 0.99) and lower blood pressure with the use of BIA [65].

A systematic review and meta-analysis conducted by the NHS on more than 5000 patients showed that BIA may lower overhydration in HD patients and improve blood pressure control, but it did not definitively prove a reduction in overall mortality [66]. The authors suggested that the difference could be due to the new studies in the meta-analysis and the increased number of peritoneal dialysis (PD) patients. Indeed, patients with residual renal function usually have a lower risk of mortality. Thus, the inclusion of more studies with PD may have led to a significant difference [66]. Differently, another recent study failed to demonstrate an improvement in short-term clinical outcomes [67]. Nevertheless, the results were not discordant in finding a significant reduction in the hospitalization rate and an impressive, although not statistically significant, trend in the improvement of the primary composite endpoint consisting of death, acute myocardial infarction, cerebral infarction, cerebral hemorrhage, and peripheral vascular disease (HR = 0.487, 95% CI 0.217–1.091, *p* = 0.08) [67,68].

**Table 2:** Comparative analysis of BIA studies in CKD patients. A selection of the studies exploring the hardest endpoints in HF patients is summarized in the table.

Author	Year	Type of Study	Patients	BIA Method	Endpoints	Limitations	Main Results
Onofriescu [60]	2014	Prospective randomized controlled trial - Hemodialysis	131	BIS	All-cause mortality	Underpowered regarding mortality outcomes due to younger cohort and a lower diabetes rate	Beneficial Effects BIA-based fluid management significantly reduced mortality in HD patients (HR = 0.100 95% CI, 0.013–0.805).
Huan-Sheng—(ABISAD III) [61]	2016	Prospective randomized controlled trial - Hemodialysis	298	BIS	All-cause hospitalizations AFO	This study focused on FO post HD; further studies are needed to show whether other interventions besides weight adjustment play a role in this improvement	Beneficial Effects AFO incidence, cardiovascular events, or intradialytic complications were significantly reduced in the intervention group. Neutral Effects All-cause hospitalization rate was not different between groups.
Zoccali [62]	2017	Prospective non-randomized study - Hemodialysis	39,566	BIS	All-cause mortality at 1 and 4 years	Purely observational nature of the study	Beneficial Effects Baseline OHI/ECW > 15% in men and >13% in women at baseline were independent predictors of mortality in HD patients (HR = 1.26, 95% CI 1.19–1.33).
Tabinor [64]	2018	Meta-analysis - Hemodialysis	60,790	Various BIA methods	Mortality	Methodological heterogeneity, inadequately reported demographics and report of endpoints	Beneficial Effects Baseline pre-dialysis OHI > 15% is predictive for mortality in HD patients (HR = 2.28, 95% CI 1.56–3.34).
Dekker [63]	2018	Prospective study - Hemodialysis	8883	BIS	Mortality	No documentation of antihypertensive medications, echocardiographic results are not available and cardiac failure is likely underreported	Beneficial Effects Pre-dialysis FO (>+1.1 to +2.5 L) together with pre-SBP < 110 mmHg was associated with an increased mortality (HR = 1.52; 95% CI 1.06–2.17).
Liu—(BOCOMO Study) [67]	2020	Prospective randomized controlled trial - Hemodialysis	445	BIS	All-cause mortality, myocardial infarction, cerebral infarction, cerebral hemorrhage, and peripheral vascular disease	Small cohort and limited follow up period	Neutral Effects An increasing trend of survival rates in patients with BIA-guided HD fluid management was observed; however, no significant difference observed (log-rank test, *p* = 0.07).
Horowitz [65]	2023	Systematic Review and Meta-Analysis - Hemodialysis	2420	Various BIA methods	All-cause mortality, blood pressure control, all-cause hospitalization, major adverse cardiovascular events, and change in left ventricular mass index	Heterogeneity in reported endpoints/outcomes	Beneficial Effects In HD patients using BIA-guided fluid management decreases all-cause mortality and blood pressure. Neutral Effects No significant difference in all-cause hospitalization, major adverse cardiac event, or change in left ventricular mass index was observed.
Stigger [68]	2023	Prospective randomized controlled trial - Hemodialysis	110	BIS	All-cause mortality, blood pressure control, and all-cause hospitalization	Small sample size	Beneficial Effects BIA-guided fluid management utilization significantly reduced the incidence rate of hospital admissions in HD patients.

AFO = average fluid overload; BIA = bioimpedance analysis; BIS = bioimpedance spectroscopy; CI = confidence interval; ECW = extracellular water; FO = fluid overload; HD = hemodialysis; HR = hazard ratio; OHI = overhydration index.

### 2.6. Diagnosis, Therapy, and Risk Stratification in HF and CKD

**Figure 1:**
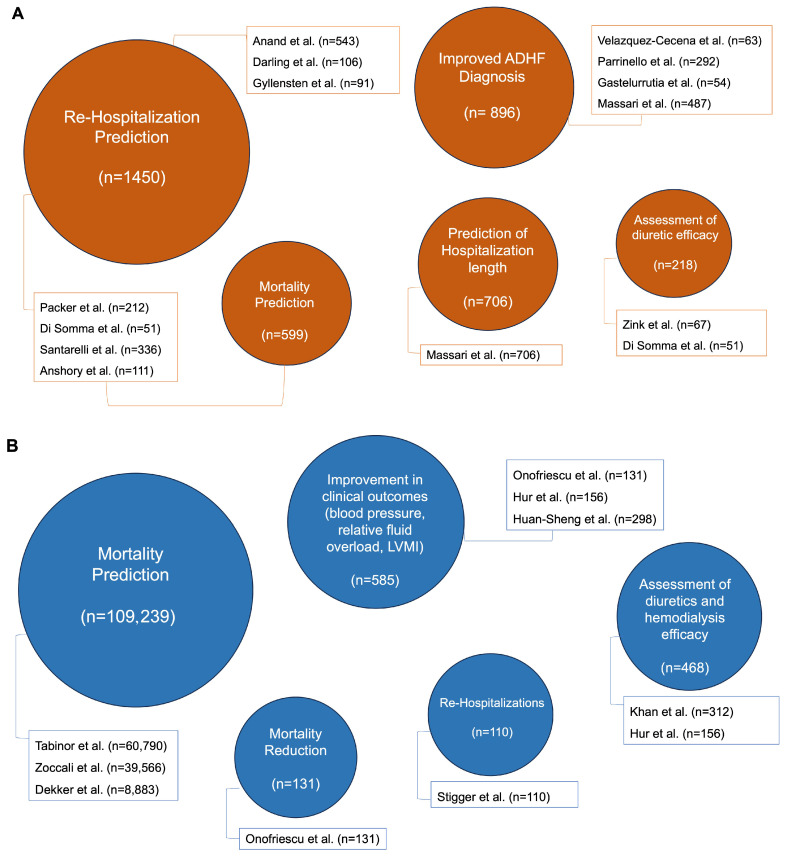
Graphical representation of the most relevant clinical results obtained with BIA in HF and CKD patients. In each figure, the most relevant clinical results obtained in the selected studies are summarized for HF (**A**) and CKD (**B**) groups. The size of the bubble is directly proportional to the number of patients in which the results have been obtained. For each parameter, the first author and the number of patients studied are shown [45,46,47,48,49,50,55,57,59,60,61,62,63,64,68,69,70,71,72,75,76]. ADHF = acute decompensated heart failure; BIA = bioimpedance analysis; CKD = chronic kidney disease; HF = heart failure; LVMI = left ventricular mass index.

BIA measurements can provide valuable information to clinicians for the diagnosis, treatment, and risk stratification of HF and CKD patients. Figure 1A,B show a visual representation of the most relevant clinical benefits of BIA for HF and CKD patients, respectively.

Various studies have repeatedly shown that BIA aids in the diagnostic assessment of the hydration status of HF and CKD patients.

Velazquez-Cecena et al. aimed to evaluate the correlation of low-, intermediate-, and high-risk groups for ADHF as determined by ICG parameters with LVEDP and serum BNP. The study’s main outcome was that patients considered at high risk for ADHF as determined by ICG presented significantly higher levels of LVEDP and BNP compared to lower-risk groups [69].

A study by Parrinello et al. was designed to evaluate the diagnostic role of segmental and whole-BIA in the differential diagnosis of ADHF from the other causes of acute dyspnea across a broad population presenting to the emergency department [70]. One of the study’s main results revealed that whole-body and segmental BIA were strong predictors of ADHF alone or in combination with BNP at the multiple regression analysis (AUC 0.989). Moreover, ROC curves were significantly higher (*p* < 0.0001) for segmental BIA (AUC = 0.963; 95% CI: 0.935–0.982) and whole-body BIA (AUC = 0.934; 95% CI: 0.902–0.961) than for LVEF (AUC = 0.814; 95% CI: 0.764–0.857) and Framingham score (AUC = 0.681; 95% CI: 0.625–0.734) [70].

In a study of ambulatory patients with HF by Gastelurrutia et al., BIVA was utilized instead to successfully differentiate between stable and unstable HF; stable patients were found to have a significantly lower impedance-measured fluid load ratio and lower nt-proBNP than the unstable ones [71].

Many studies concentrated on the role of BIA to guide and assess the efficacy of diuretic therapy in ADHF.

In a study by Zink et al., segmental BIA measurement was employed for the in-hospital monitoring of cardiac re-compensation in HF patients, which revealed the location of fluid accumulation and shift within the body during diuretic treatment [72].

In a study by Di Somma et al., the authors demonstrated a direct correlation between BIVA and urine output at 72 h, showing how the efficacy of diuretic therapy and the effectiveness of these measurements go hand in hand with the normalization of BNP and caval index values at discharge. Thus, the authors suggested that BIVA could be more useful than clinical signs in managing diuretic therapy in ADHF patients [46].

Concerning CKD, a study aimed to evaluate the clinical usefulness of BIA in assessing the volume status in patients receiving maintenance dialysis. The results indicated that BIA can accurately assess the fluid volume status in patients receiving maintenance dialysis as measured by ECW, and it can predict volume overload and hypertension in patients with end-stage renal disease. Lastly, the study indicated that BIA can be used to monitor changes in volume status over time and to guide clinical decision making in managing fluid volume in patients receiving maintenance dialysis [73].

The group of Khan et al. demonstrated the efficacy of BIS in the stratification of CKD patients based on their volemic status and therefore randomized them to different diuretic therapy approaches, with good results compared to controls [57].

Detecting body fluids congestion in HF is challenging, and many patients are discharged from hospital in a still-congested state [74,75]. In a study on hospitalized ADHF patients, Massari et al. demonstrated that BIVA could predict the total length of hospital stay independently. In other words, the more congested the patients were, the longer their stay in hospital [75].

Other studies investigated the role of BIA (i.e., BIVA) and BNP or NTproBNP for the risk stratification of HF patients [46,76,77]. In a study by Massari et al., BIVA was more accurate than BNP in the detection of peripheral congestion in both ADHF (AUC was 0.88 vs. 0.57, respectively; *p* < 0.001) and in chronic HF (AUC was 0.89 vs. 0.68, respectively; *p* < 0.001). Algorithms that include BIVA together with NTproBNP or BNP were shown to be extremely accurate for patients’ prognosis assessment [76].

Eventually, a meta-analysis on the role of BIA (leg bioimpedance) in relation to incident HF in the general population, with more than 500,000 patients, was presented by Lindholm et al. in 2018 [78]. This multivariable model, which included leg bioimpedance, age, sex, and self-reported prior myocardial infarction, showed good discrimination for future HF hospitalization, highlighting the potential benefits of adopting this simple, non-invasive, and cost-effective measure [78].

### 2.7. Advantages of BIA

In this narrative review, we presented a generalized beneficial effect of BIA for both HF and CKD patients in predicting hard endpoints such as ADHF, re-hospitalizations, and mortality.

**Figure 2:**
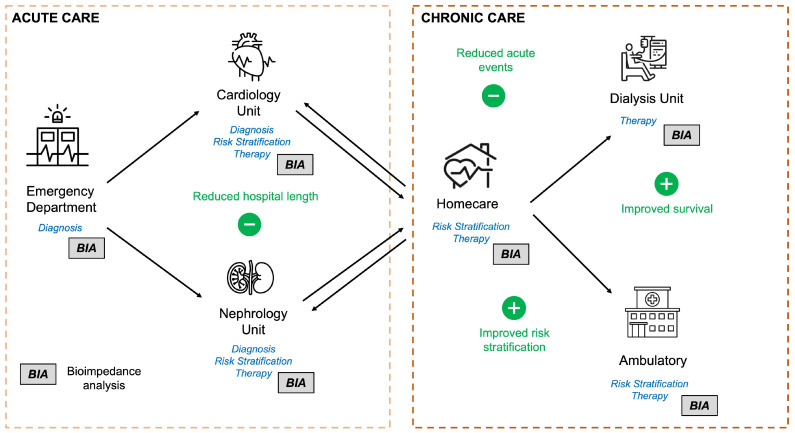
Graphical representation of the proven clinical benefits of BIA in acute and chronic care settings. The usual clinical course of CKD and HF patients from acute to chronic care is displayed. The studied role of BIA for diagnosis, risk stratification, and therapy in each setting are written in blue. The clinical benefit of using BIA in each setting is shown in green. BIA = bioimpedance analysis; CKD = chronic kidney disease; HF = heart failure.

BIA is a safe and affordable technique whose utilization is not operator-dependent due to its highly proven accuracy and repeatability [11]. Due to its non-invasiveness, it can be adapted to different clinical settings [11]. Figure 2 depicts the potential clinical benefits of utilizing BIA in acute and chronic care settings.

Moreover, BIA has been shown to provide many advantages, and its utilization can theoretically span from the emergency room to the bedside clinical ward assessment up to remote monitoring.

While we currently describe its role in the assessment of hydration, a great amount of evidence is also present regarding the role of BIA in assessing the nutrition status of chronic patients [11].

### 2.8. Limitations of BIA

Even if the advantages of BIA are clear, the technique has some limitations. Body composition assessment through BIA is highly reproducible in ideal conditions. However, the correct positioning and adherence of the electrodes to the skin need to be considered a potential confounding factor. A supine condition with a hand-to-foot configuration is considered the most accurate as it estimates the whole-body surface but may be difficult to obtain with certain patients, especially if they are acutely decompensated or undergoing an HD procedure [79].

One of the main limitations is that BIA mostly uses regression models derived from healthy people to predict body composition [80]. While BIA has been validated in conditions such as HF and CKD and in different population subsets, it is relevant to constantly study and adapt novel dedicated equations for an accurate estimation of the body composition in specific pathological conditions [11]. For instance, BIA is inaccurate for morbidly obese patients because an excess of adipose tissue can overestimate FFM while underestimating FM values. This is mainly caused by the different distribution between body mass and body conductivity, reducing the accuracy detection of BIA [11,17].

CKD is associated with fluid retention, which increases total body water (TBW) and leads to changes in intracellular water (ICW) and ECW. Other confounders such as ethnicity, gender, body mass index, and estimated glomerular filtration rate, may affect BIA accuracy and outcome prediction [11].

A theoretical limitation of BIA regards its utilization in patients carrying cardiac implantable electronic devices (CIEDs) due to potential electromagnetic interference. Nonetheless, different studies approached the problem, showing that neither BIA nor the implantable devices interfered with or provided any risks to the functioning and measurement of the other method [81].

While this current review does not concentrate on the analysis of the costs of BIA equipment and usage, it is relevant to state that BIA is not a regularly reimbursed medical product, limiting its utilization in everyday settings or smaller centers with fewer economical resources.

### 2.9. Future Perspectives

Although the clinical relevance of BIA has been repeatedly demonstrated in recent years, its everyday utilization in clinical wards or for remote monitoring remains limited.

One of the main problems regarding BIA adoption is the lack of large, randomized trials that could justify a complete reimbursement of the solution. Further studies should question the benefits of BIA, comparing the scenarios in which it is employed against those in which it is not applied, with the hypothesis that it can improve the current standard of care. This holds true for in-hospital use, but it is even more relevant for home settings.

Another point that needs to be addressed when considering its adoption is the complete integration of the measurement of HS into the clinical work routine.

Currently, the assessment of HS in a hospital is still mostly based on physical examination by the medical doctor, while patients use only weight scales at home. Therefore, the detection of HS should be accessible not only at the patient’s bedside but also at the patient’s home.

Hydration status should be an easily visualizable and actionable parameter, like blood pressure, blood glucose, or temperature, and channeled into a clinical decision tree.

Thus, it is crucial to have dedicated and instructed personnel collecting and interpreting the information. However, future solutions should focus on automatizing the information workflow and introducing smart sensors.

A recent study showed that a handheld bioimpedance system for the assessment of body fluid status could be used to assist the patient in home-based ESKD treatments [82]. This device consisted of a custom-made handheld tetrapolar bioimpedance spectrometer and a textile-based electrode garment for total body fluid assessment [82]. Future solutions should follow those developments.

We believe optimizing processes and improving clinical outcomes should drive HS adoption, with patients’ needs as the focus of our attention.

Another extremely relevant aspect concerns the creation of personalized pathology parameters that, through AI and the analysis of large datasets, should guide the personalization of treatments. Indeed, combining different biomarkers with BIA has been shown to be very promising for prognostic purposes. For this reason, our study group recently finalized a study currently under revision to assess the role of BIA, biomarkers, and other vital parameters to create a risk prediction algorithm that could detect mortality and re-hospitalizations within 6 months.

## 3. Conclusions

The importance of BIA in detecting and predicting the clinical course of patients has been demonstrated. The fundamental step of integrating hydration status assessment into the everyday routine for the management of CKD and HF patients is needed. Further randomized trials are needed to prove that using BIA compared to standard care may reduce re-hospitalizations and mortality, especially in remote monitoring settings.

## Data Availability

No new data were created.

## References

[1] National Institutes of Health (1996). Bioelectrical impedance analysis in body composition measurement: National Institutes of Health Technology Assessment Conference Statement. Am. J. Clin. Nutr..

[2] Lukaski H.C., Bolonchuk W.W., Hall C.B., Siders W.A. (1986). Validation of tetrapolar bioelectrical impedance method to assess human body composition. J. Appl. Physiol..

[3] Lukaski H.C., Vega Diaz N., Talluri A., Nescolarde L. (2019). Classification of Hydration in Clinical Conditions: Indirect and Direct Approaches Using Bioimpedance. Nutrients.

[4] Lukaski H.C., Talluri A. (2023). Phase angle as an index of physiological status: Validating bioelectrical assessments of hydration and cell mass in health and disease. Rev. Endocr. Metab. Disord..

[5] Baumgartner R.N., Chumlea W.C., Roche A. (1989). Estimation of body composition from bioelectric impedance of body segments. Am. J. Clin. Nutr..

[6] Kotanko P., Levin N.W., Zhu F. (2008). Current state of bioimpedance technologies in dialysis. Nephrol. Dial. Transplant..

[7] Thomas B.J., Ward L.C., Cornish B.H. (1998). Bioimpedance spectrometry in the determination of body water compartments: Accuracy and clinical significance. Appl. Radiat. Isot..

[8] Chamney P.W., Krämer M., Rode C., Kleinekofort W., Wizemann V. (2002). A new technique for establishing dry weight in hemodialysis patients via whole body bioimpedance. Kidney Int..

[9] Charra B. (2007). Fluid balance, dry weight, and blood pressure in dialysis. Hemodial. Int..

[10] Khalil S.F., Mohktar M.S., Ibrahim F. (2014). The theory and fundamentals of bioimpedance analysis in clinical status monitoring and diagnosis of diseases. Sensors.

[11] Kyle U.G., Bosaeus I., De Lorenzo A.D., Deurenberg P., Elia M., Gómez J.M., Heitmann B.L., Kent-Smith L., Melchior J.-C., Pirlich M. (2004). Bioelectrical impedance analysis—Part I: Review of principles and methods. Clin. Nutr..

[12] La Porta E., Lanino L., Calatroni M., Caramella E., Avella A., Quinn C., Faragli A., Estienne L., Alogna A., Esposito P. (2021). Volume Balance in Chronic Kidney Disease: Evaluation Methodologies and Innovation Opportunities. Kidney Blood Press. Res..

[13] Ronco C., Haapio M., House A.A., Anavekar N., Bellomo R. (2008). Cardiorenal syndrome. J. Am. Coll. Cardiol..

[14] de Jager D.J., Grootendorst D.C., Jager K.J., van Dijk P.C., Tomas L.M., Ansell D., Collart F., Finne P., Heaf J.G., De Meester J. (2009). Cardiovascular and noncardiovascular mortality among patients starting dialysis. JAMA.

[15] Deferrari G., Cipriani A., La Porta E. (2021). Renal dysfunction in cardiovascular diseases and its consequences. J. Nephrol..

[16] Faragli A., La Porta E., Campana C., Pieske B., Kelle S., Koehler F., Alogna A. (2020). Out-of-Hospital Care of Heart Failure Patients During and After COVID-19 Pandemic: Time for Telemedicine?. Front. Digit. Health.

[17] Kyle U.G., Bosaeus I., De Lorenzo A.D., Deurenberg P., Elia M., Manuel Gómez J., Heitmann B.L., Kent-Smith L., Melchior J.-C., Pirlich M. (2004). Bioelectrical impedance analysis—Part II: Utilization in clinical practice. Clin. Nutr..

[18] Hoffer E.C., Meador C.K., Simpson D.C. (1969). Correlation of whole-body impedance with total body water volume. J. Appl. Physiol..

[19] Lukaski H.C. (2013). Evolution of bioimpedance: A circuitous journey from estimation of physiological function to assessment of body composition and a return to clinical research. Eur. J. Clin. Nutr..

[20] Bera T.K. (2014). Bioelectrical impedance methods for noninvasive health monitoring: A review. J. Med. Eng..

[21] Keren H., Burkhoff D., Squara P. (2007). Evaluation of a noninvasive continuous cardiac output monitoring system based on thoracic bioreactance. Am. J. Physiol.-Heart Circ. Physiol..

[22] Wang L. (2007). Fundamentals of intrathoracic impedance monitoring in heart failure. Am. J. Cardiol..

[23] Yang X.-W., Hua W., Ding L.-G., Wang J., Zheng L.-H., Li C.-Q., Liu Z.-M., Chen K.-P., Zhang S. (2013). OptiVol fluid index predicts acute decompensation of heart failure with a high rate of unexplained events. J. Geriatr. Cardiol. JGC.

[24] Tang W.H., Tong W. (2009). Measuring impedance in congestive heart failure: Current options and clinical applications. Am. Heart J..

[25] Yu C.-M., Wang L., Chau E., Chan R.H.-W., Kong S.-L., Tang M.-O., Christensen J., Stadler R.W., Lau C.-P. (2005). Intrathoracic impedance monitoring in patients with heart failure: Correlation with fluid status and feasibility of early warning preceding hospitalization. Circulation.

[26] Felker G.M., Lee K.L., Bull D.A., Redfield M.M., Stevenson L.W., Goldsmith S.R., LeWinter M.M., Deswal A., Rouleau J.L., Ofili E.O. (2011). Diuretic strategies in patients with acute decompensated heart failure. N. Engl. J. Med..

[27] Savarese G., Lund L.H. (2017). Global public health burden of heart failure. Card. Fail. Rev..

[28] Roger V.L. (2013). Epidemiology of heart failure. Circ. Res..

[29] Ponikowski P., Voors A.A., Anker S.D., Bueno H., Cleland J.G., Coats A.J., Falk V., González-Juanatey J.R., Harjola V.-P., Jankowska E.A. (2016). 2016 ESC Guidelines for the diagnosis and treatment of acute and chronic heart failure: The Task Force for the diagnosis and treatment of acute and chronic heart failure of the European Society of Cardiology (ESC). Developed with the special contribution of the Heart Failure Association (HFA) of the ESC. Eur. J. Heart Fail..

[30] Boorsma E.M., ter Maaten J.M., Damman K., Dinh W., Gustafsson F., Goldsmith S., Burkhoff D., Zannad F., Udelson J.E., Voors A.A. (2020). Congestion in heart failure: A contemporary look at physiology, diagnosis and treatment. Nat. Rev. Cardiol..

[31] Ishikawa S.-e. (2015). Hyponatremia associated with heart failure: Pathological role of vasopressin-dependent impaired water excretion. J. Clin. Med..

[32] Palazzuoli A., Evangelista I., Nuti R. (2020). Congestion occurrence and evaluation in acute heart failure scenario: Time to reconsider different pathways of volume overload. Heart Fail. Rev..

[33] Gheorghiade M., Filippatos G., De Luca L., Burnett J. (2006). Congestion in acute heart failure syndromes: An essential target of evaluation and treatment. Am. J. Med..

[34] Pellicori P., Kaur K., Clark A.L. (2015). Fluid Management in Patients with Chronic Heart Failure. Card. Fail. Rev..

[35] O’Connor C.M., Stough W.G., Gallup D.S., Hasselblad V., Gheorghiade M. (2005). Demographics, clinical characteristics, and outcomes of patients hospitalized for decompensated heart failure: Observations from the IMPACT-HF registry. J. Card. Fail..

[36] Neuberg G.W., Miller A.B., O’Connor C.M., Belkin R.N., Carson P.E., Cropp A.B., Frid D.J., Nye R.G., Pressler M.L., Wertheimer J.H. (2002). Diuretic resistance predicts mortality in patients with advanced heart failure. Am. Heart J..

[37] Lewin J., Ledwidge M., O’Loughlin C., McNally C., McDonald K. (2005). Clinical deterioration in established heart failure: What is the value of BNP and weight gain in aiding diagnosis?. Eur. J. Heart Fail..

[38] Chaudhry S.I., Mattera J.A., Curtis J.P., Spertus J.A., Herrin J., Lin Z., Phillips C.O., Hodshon B.V., Cooper L.S., Krumholz H.M. (2010). Telemonitoring in patients with heart failure. N. Engl. J. Med..

[39] Evangelista L.S., Lee J.A., Moore A.A., Motie M., Ghasemzadeh H., Sarrafzadeh M., Mangione C.M.M. (2015). Examining the effects of remote monitoring systems on activation, self-care, and quality of life in older patients with chronic heart failure. J. Cardiovasc. Nurs..

[40] Faragli A., Abawi D., Quinn C., Cvetkovic M., Schlabs T., Tahirovic E., Düngen H.-D., Pieske B., Kelle S., Edelmann F. (2021). The role of non-invasive devices for the telemonitoring of heart failure patients. Heart Fail. Rev..

[41] Abraham W.T., Adamson P.B., Bourge R.C., Aaron M.F., Costanzo M.R., Stevenson L.W., Strickland W., Neelagaru S., Raval N., Krueger S. (2011). Wireless pulmonary artery haemodynamic monitoring in chronic heart failure: A randomised controlled trial. Lancet.

[42] Vaduganathan M., DeFilippis E.M., Fonarow G.C., Butler J., Mehra M.R. (2017). Postmarketing Adverse Events Related to the CardioMEMS HF System. JAMA Cardiol..

[43] Singh R., Varjabedian L., Kaspar G., Zughaib M. (2018). CardioMEMS in a Busy Cardiology Practice: Less than Optimal Implementation of a Valuable Tool to Reduce Heart Failure Readmissions. Cardiol. Res. Pract..

[44] Koehler F., Koehler K., Deckwart O., Prescher S., Wegscheider K., Winkler S., Vettorazzi E., Polze A., Stangl K., Hartmann O. (2018). Telemedical Interventional Management in Heart Failure II (TIM-HF2), a randomised, controlled trial investigating the impact of telemedicine on unplanned cardiovascular hospitalisations and mortality in heart failure patients: Study design and description of the intervention. Eur. J. Heart Fail..

[45] Packer M., Abraham W.T., Mehra M.R., Yancy C.W., Lawless C.E., Mitchell J.E., Smart F.W., Bijou R., O’Connor C.M., Massie B.M. (2006). Utility of impedance cardiography for the identification of short-term risk of clinical decompensation in stable patients with chronic heart failure. J. Am. Coll. Cardiol..

[46] Di Somma S., De Berardinis B., Bongiovanni C., Marino R., Ferri E., Alfei B. (2010). Use of BNP and bioimpedance to drive therapy in heart failure patients. Congest. Heart Fail..

[47] Santarelli S., Russo V., Lalle I., De Berardinis B., Vetrone F., Magrini L., Di Stasio E., Piccoli A., Codognotto M., Mion M.M. (2017). Prognostic value of decreased peripheral congestion detected by Bioelectrical Impedance Vector Analysis (BIVA) in patients hospitalized for acute heart failure: BIVA prognostic value in acute heart failure. Eur. Heart J. Acute Cardiovasc. Care.

[48] Anand I.S., Tang W.W., Greenberg B.H., Chakravarthy N., Libbus I., Katra R.P., Music Investigators (2012). Design and performance of a multisensor heart failure monitoring algorithm: Results from the multisensor monitoring in congestive heart failure (MUSIC) study. J. Card. Fail..

[49] Gyllensten I.C., Bonomi A.G., Goode K.M., Reiter H., Habetha J., Amft O., Cleland J.G. (2016). Early indication of decompensated heart failure in patients on home-telemonitoring: A comparison of prediction algorithms based on daily weight and noninvasive transthoracic bio-impedance. JMIR Med. Inform..

[50] Darling C.E., Dovancescu S., Saczynski J.S., Riistama J., Kuniyoshi F.S., Rock J., Meyer T.E., McManus D.D. (2017). Bioimpedance-Based Heart Failure Deterioration Prediction Using a Prototype Fluid Accumulation Vest-Mobile Phone Dyad: An Observational Study. JMIR Cardio.

[51] Wang L., Lahtinen S., Lentz L., Rakow N., Kaszas C., Ruetz L., Stylos L., Olson W.H. (2005). Feasibility of using an implantable system to measure thoracic congestion in an ambulatory chronic heart failure canine model. Pacing Clin. Electrophysiol..

[52] van Veldhuisen D.J., Braunschweig F., Conraads V., Ford I., Cowie M.R., Jondeau G., Kautzner J., Aguilera R.M., Lunati M., Yu C.M. (2011). Intrathoracic impedance monitoring, audible patient alerts, and outcome in patients with heart failure. Circulation.

[53] Anshory M., Kuan W.S., Rohman M.S., Waranugraha Y., Kamila P.A., Iskandar A., Susianti H., Yau Y.W., Soh C.H.W., Ali K.M. (2024). Can non-invasive cardiac hemodynamics and fluid content system (NICaS) parameters predict Acute Heart Failure outcomes in Caucasian and Asian patients in the emergency department?. Adv. Med. Sci..

[54] McCullough K., Sharma P., Ali T., Khan I., Smith W.C., MacLeod A., Black C. (2012). Measuring the population burden of chronic kidney disease: A systematic literature review of the estimated prevalence of impaired kidney function. Nephrol. Dial. Transplant..

[55] McCullough P.A., Chan C.T., Weinhandl E.D., Burkart J.M., Bakris G.L. (2016). Intensive Hemodialysis, Left Ventricular Hypertrophy, and Cardiovascular Disease. Am. J. Kidney Dis..

[56] Soi V., Yee J. (2017). Sodium Homeostasis in Chronic Kidney Disease. Adv. Chronic Kidney Dis..

[57] Khan Y.H., Sarriff A., Adnan A.S., Khan A.H., Mallhi T.H. (2017). Diuretics prescribing in chronic kidney disease patients: Physician assessment versus bioimpedence spectroscopy. Clin. Exp. Nephrol..

[58] Davies S.J., Caskey F.J., Coyle D., Lindley E., Macdonald J., Mitra S., Wilkie M., Davenport A., Farrington K., Dasgupta I. (2017). Rationale and design of BISTRO: A randomized controlled trial to determine whether bioimpedance spectroscopy-guided fluid management maintains residual kidney function in incident haemodialysis patients. BMC Nephrol..

[59] Hur E., Usta M., Toz H., Asci G., Wabel P., Kahvecioglu S., Kayikcioglu M., Demirci M.S., Ozkahya M., Duman S. (2013). Effect of fluid management guided by bioimpedance spectroscopy on cardiovascular parameters in hemodialysis patients: A randomized controlled trial. Am. J. Kidney Dis..

[60] Onofriescu M., Hogas S., Voroneanu L., Apetrii M., Nistor I., Kanbay M., Covic A.C. (2014). Bioimpedance-guided fluid management in maintenance hemodialysis: A pilot randomized controlled trial. Am. J. Kidney Dis..

[61] Huan-Sheng C., Yeong-Chang C., Ming-Hsing H., Fan-Lieh T., Chu-Cheng L., Tsai-Kun W., Hung-Ping C., Sze-Hung H., Hsien-Chang C., Chia-Chen L. (2016). Application of bioimpedance spectroscopy in Asian dialysis patients (ABISAD-III): A randomized controlled trial for clinical outcomes. Int. Urol. Nephrol..

[62] Zoccali C., Moissl U., Chazot C., Mallamaci F., Tripepi G., Arkossy O., Wabel P., Stuard S. (2017). Chronic Fluid Overload and Mortality in ESRD. J. Am. Soc. Nephrol..

[63] Dekker M., Konings C., Canaud B., Carioni P., Guinsburg A., Madero M., van der Net J., Raimann J., van der Sande F., Stuard S. (2018). Pre-dialysis fluid status, pre-dialysis systolic blood pressure and outcome in prevalent haemodialysis patients: Results of an international cohort study on behalf of the MONDO initiative. Nephrol. Dial. Transpl. Transplant..

[64] Tabinor M., Elphick E., Dudson M., Kwok C.S., Lambie M., Davies S.J. (2018). Bioimpedance-defined overhydration predicts survival in end stage kidney failure (ESKF): Systematic review and subgroup meta-analysis. Sci. Rep..

[65] Horowitz L., Karadjian O., Braam B., Mavrakanas T., Weber C. (2023). Bioimpedance-Guided Monitoring of Volume Status in Patients With Kidney Disease: A Systematic Review and Meta-Analysis. Can. J. Kidney Health Dis..

[66] Scotland G., Cruickshank M., Jacobsen E., Cooper D., Fraser C., Shimonovich M., Marks A., Brazzelli M. (2018). Multiple-frequency bioimpedance devices for fluid management in people with chronic kidney disease receiving dialysis: A systematic review and economic evaluation. Health Technol. Assess..

[67] Liu L., Sun Y., Chen Y., Xu J., Yuan P., Shen Y., Lin S., Sun W., Ma Y., Ren J. (2020). The effect of BCM guided dry weight assessment on short-term survival in Chinese hemodialysis patients: Primary results of a randomized trial—BOdy COmposition MOnitor (BOCOMO) study. BMC Nephrol..

[68] Stigger K., Ribeiro L.R., Cordeiro F.M., Böhlke M. (2023). Incidence of hospital admissions in bioimpedance-guided fluid management among maintenance hemodialysis patients-Results of a randomized controlled trial. Hemodial. Int..

[69] Velazquez-Cecena J.L., Sharma S., Nagajothi N., Khraisat A., Khosla S., Arora R.R., Benatar D. (2008). Left ventricular end diastolic pressure and serum brain natriuretic peptide levels in patients with abnormal impedance cardiography parameters. Arch. Med. Res..

[70] Parrinello G., Paterna S., Di Pasquale P., Torres D., Fatta A., Mezzero M., Scaglione R., Licata G. (2008). The usefulness of bioelectrical impedance analysis in differentiating dyspnea due to decompensated heart failure. J. Card. Fail..

[71] Gastelurrutia P., Nescolarde L., Rosell-Ferrer J., Domingo M., Ribas N., Bayes-Genis A. (2011). Bioelectrical impedance vector analysis (BIVA) in stable and non-stable heart failure patients: A pilot study. Int. J. Cardiol..

[72] Zink M.D., König F., Weyer S., Willmes K., Leonhardt S., Marx N., Napp A. (2020). Segmental bioelectrical impedance spectroscopy to monitor fluid status in heart failure. Sci. Rep..

[73] Park J.H., Jo Y.I., Lee J.H. (2018). Clinical usefulness of bioimpedance analysis for assessing volume status in patients receiving maintenance dialysis. Korean J. Intern. Med..

[74] Maddox T.M., Januzzi J.L., Allen L.A., Breathett K., Butler J., Davis L.L., Fonarow G.C., Ibrahim N.E., Lindenfeld J., Masoudi F.A. (2021). 2021 Update to the 2017 ACC Expert Consensus Decision Pathway for Optimization of Heart Failure Treatment: Answers to 10 Pivotal Issues About Heart Failure With Reduced Ejection Fraction: A Report of the American College of Cardiology Solution Set Oversight Committee. J. Am. Coll. Cardiol..

[75] Massari F., Scicchitano P., Ciccone M.M., Caldarola P., Aspromonte N., Iacoviello M., Barro S., Pantano I., Valle R. (2019). Bioimpedance vector analysis predicts hospital length of stay in acute heart failure. Nutrition.

[76] Massari F., Iacoviello M., Scicchitano P., Mastropasqua F., Guida P., Riccioni G., Speziale G., Caldarola P., Ciccone M.M., Di Somma S. (2016). Accuracy of bioimpedance vector analysis and brain natriuretic peptide in detection of peripheral edema in acute and chronic heart failure. Heart Lung.

[77] Massari F., Scicchitano P., Iacoviello M., Passantino A., Guida P., Sanasi M., Piscopo A., Romito R., Valle R., Caldarola P. (2020). Multiparametric approach to congestion for predicting long-term survival in heart failure. J. Cardiol..

[78] Lindholm D., Fukaya E., Leeper N.J., Ingelsson E. (2018). Bioimpedance and new-onset heart failure: A longitudinal study of> 500 000 individuals from the general population. J. Am. Heart Assoc..

[79] Montalibet A., Rastel D., Chaigneau C., Grenier E., McAdams E. (2020). Comparison between bioelectrical impedance spectroscopy measurements and water volume displacement of ankle oedema variations during the course of a day. Physiol. Meas..

[80] Sun S.S., Chumlea W.C., Heymsfield S.B., Lukaski H.C., Schoeller D., Friedl K., Kuczmarski R.J., Flegal K.M., Johnson C.L., Hubbard V.S. (2003). Development of bioelectrical impedance analysis prediction equations for body composition with the use of a multicomponent model for use in epidemiologic surveys. Am. J. Clin. Nutr..

[81] Chabin X., Taghli-Lamallem O., Mulliez A., Bordachar P., Jean F., Futier E., Massoullié G., Andonache M., Souteyrand G., Ploux S. (2019). Bioimpedance analysis is safe in patients with implanted cardiac electronic devices. Clin. Nutr..

[82] Ferreira J., Pau I., Lindecrantz K., Seoane F. (2017). A Handheld and Textile-Enabled Bioimpedance System for Ubiquitous Body Composition Analysis. An Initial Functional Validation. IEEE J. Biomed. Health Inform..

